# Host determinants of immune-related adverse events during immune checkpoint inhibition

**DOI:** 10.3389/fphar.2026.1941544

**Published:** 2026-10-14

**Authors:** Akram Alwithenani

**Affiliations:** Department of Clinical Laboratory Sciences, Umm Al-Qura University, Makkah, Saudi Arabia

**Keywords:** gut microbiome, immune checkpoint inhibitors, immune-related adverse events, immuno-oncology, predictive biomarkers

## Abstract

Immune checkpoint inhibitors (ICIs) have transformed the treatment of many cancers, but immune-related adverse events (irAEs) have become their principal limitation. This review argues that irAEs are not a random hazard of checkpoint blockade but a largely host-determined outcome layered on a drug-set baseline. The regimen fixes how much toxicity risk exists and, through the checkpoint it blocks, which organs are most likely affected. Within that envelope, host determinants decide which patient crosses the threshold of tolerance, and where. We synthesise the evidence for four classes of host determinant: germline genetic variation, including the IL7 locus and organ-directing human leucocyte antigen (HLA) alleles; the baseline immune landscape; the gut microbiome and metabolic state; and clinical characteristics led by pre-existing autoimmune disease. We draw two distinctions the literature often blurs. First, susceptibility should be read in two stages: determinants measurable before the first dose, which can triage patients, and on-treatment changes, which can only signal an event already emerging. Second, organ-specific toxicity is neither drug- nor host-intrinsic but the product of their intersection, with HLA restriction and shared tumour-tissue antigens offering the clearest mechanistic account of why a particular organ is struck. One theme runs throughout: many host features that raise irAE risk also predict a better antitumour response, so host stratification is a matter of optimising a benefit-to-toxicity balance rather than screening susceptible patients out. Multi-omics and systems immunology are assembling the measurements needed to act on this biology, but no host-based pre-treatment predictor is yet validated for clinical use. Realising one would shift checkpoint immunotherapy from the reactive management of toxicity toward its anticipation.

## 1 Introduction

Immune checkpoint inhibitors (ICIs) have redefined the treatment of many advanced malignancies. Antibodies targeting cytotoxic T-lymphocyte antigen 4 (CTLA-4), programmed cell death protein 1 (PD-1), and its ligand PD-L1 produce durable responses across melanoma, non-small-cell lung cancer, renal cell carcinoma, and an expanding range of other tumours (Hodi et al., 2010; Pardoll, 2012). The mechanism that drives these responses carries an intrinsic cost. By releasing the inhibitory signals that normally restrain T-cell activation, ICIs can break self-tolerance and provoke immune-related adverse events (irAEs) that affect virtually any organ system, ranging from transient and low-grade to chronic, disabling, or fatal (Postow et al., 2018; Ramos-Casals et al., 2020).

IrAEs are now the principal limitation of checkpoint blockade, yet their occurrence is strikingly uneven, and two sources of that unevenness are worth separating at the outset because they have different origins. The first is attributable to the therapy itself. The intensity and pattern of toxicity scale with the regimen: combination CTLA-4 and PD-1 blockade produces grade 3 or 4 treatment-related events in roughly 55% of patients, compared with about 16% for PD-1 monotherapy, a more than threefold difference driven by the drugs rather than the patient (Larkin et al., 2015). Toxicity also carries a class signature, with anti-CTLA-4 agents disproportionately associated with colitis and hypophysitis and anti-PD-1 agents skewing toward pneumonitis, thyroid dysfunction, and vitiligo (Khoja et al., 2017). Which checkpoint is blocked, and whether one or two are blocked, therefore sets a baseline level and organ distribution of risk before any individual characteristic is considered.

The second source of unevenness is not explained by the drug. Among patients receiving the same agent at the same dose for the same tumour, only a fraction develop irAEs, the affected organ differs from person to person, and severity spans the full clinical range. This residual variation is the clinically consequential one, because it is precisely what a clinician cannot predict at the point of prescribing. It implies that the regimen defines the envelope of possible toxicity, while something intrinsic to the individual determines who, within that envelope, is affected and where.

This review develops that second idea. We argue that, once the regimen-defined baseline is accounted for, the development, organ specificity, and severity of irAEs are shaped substantially by host determinants: inherited genetic variation, the composition and prior activation state of the immune system, the microbiome, metabolic status, and a range of clinical characteristics. Rather than cataloguing organ-specific toxicities or their management, which existing reviews already address in depth (Postow et al., 2018; Ramos-Casals et al., 2020), we focus on why susceptibility varies between individuals and on how that variation might be measured before treatment begins. Framed this way, irAE risk becomes a problem of host stratification, and the tools of multi-omics and systems immunology become directly relevant to it. What follows examines each class of host determinant, the technologies used to integrate them, the host basis of organ-specific toxicity, and the prospects for translating this understanding into pre-treatment risk prediction. This is a narrative review: it synthesises the recent literature on host-level determinants of irAEs, prioritising primary human studies and foundational mechanistic work, to build an integrative framework rather than an exhaustive systematic survey.

## 2 Immune tolerance as the foundation of immune-related adverse events

If irAEs are a breakdown of self-tolerance, then the mechanisms that normally maintain that tolerance are the right starting point for understanding why the breakdown varies between patients. Immunological self-tolerance is built in two layers. Central tolerance, in the thymus, deletes or diverts the most strongly self-reactive T cells during development, but it is deliberately incomplete, and a repertoire of weakly self-reactive T cells escapes to the periphery in every healthy individual. Peripheral tolerance exists to restrain those cells, and it is here that checkpoint pathways do their work. Regulatory T (Treg) cells, inhibitory receptors, and the control of costimulation together hold self-reactive clones below the threshold of activation, and the integrity of this peripheral layer, rather than the completeness of thymic selection, is what checkpoint blockade puts under strain (Sakaguchi et al., 2020).

CTLA-4 and PD-1 occupy different points in this peripheral system, and the distinction matters for what follows. CTLA-4 acts early, during T-cell priming in lymphoid tissue. It is constitutively expressed on Tregs and induced on conventional T cells after activation, and it outcompetes the costimulatory receptor CD28 for their shared ligands CD80 and CD86, in part by physically removing those ligands from antigen-presenting cells through trans-endocytosis (Qureshi et al., 2011; Walker and Sansom, 2011). Its non-redundancy is stark: mice lacking CTLA-4 die within three to four weeks of birth from systemic lymphoproliferation and multiorgan destruction (Waterhouse et al., 1995; Tivol et al., 1995), and deleting CTLA-4 only in Tregs reproduces fatal autoimmunity, showing that the molecule enforces tolerance largely through the regulatory compartment (Wing et al., 2008). PD-1 acts later and more locally, during the effector phase in peripheral tissues, where its ligand PD-L1 is expressed broadly on non-lymphoid tissue and restrains already-activated T cells at the site of a response (Keir et al., 2008). The phenotype of its loss is correspondingly milder and, tellingly, organ-restricted: PD-1-deficient mice develop delayed, tissue-specific autoimmunity rather than a systemic catastrophe (Nishimura et al., 1999; Nishimura et al., 2001).

That contrast carries a lesson that anticipates the rest of this review. The two checkpoints do not merely differ in severity. The organ targeted by PD-1 loss depends on the genetic background of the mouse, producing lupus-like glomerulonephritis and arthritis on one strain and autoimmune cardiomyopathy on another (Nishimura et al., 1999; Nishimura et al., 2001). The same molecular lesion selects different target organs in different hosts. Even in a controlled knockout, the checkpoint sets the stage while host genotype selects the scene.

Checkpoint inhibition in patients reproduces this biology pharmacologically. Blocking CTLA-4 or PD-1 does not create novel autoreactivity so much as lower the threshold at which pre-existing self-reactive clones become active, releasing brakes that were holding a repertoire every person already carries. The predictable consequences follow: expansion of autoreactive T cells, help for autoantibody-producing B cells, and inflammatory tissue damage that presents clinically as an irAE. Because CTLA-4 blockade lifts restraint at the priming stage across the lymphoid system while PD-1 blockade lifts it at the effector stage in tissues, the two produce the distinct toxicity signatures noted earlier, and combining them removes both brakes at once (Larkin et al., 2015; Khoja et al., 2017).

This yields the conceptual frame for the review. An irAE is a pharmacologically induced, regionally expressed failure of peripheral tolerance. The drug determines how far the tolerance threshold is lowered and, through its point of action, which stage and compartment are affected. But whether that lowered threshold is actually crossed, in whom, and in which organ, depends on how much tolerance margin the individual had to begin with: the fitness of their Treg compartment, the size and specificity of their autoreactive repertoire, the inflammatory state of their tissues, and the genetic and environmental factors that set all three. Those host-side variables are the subject of what follows.

## 3 Host determinants of susceptibility to immune-related adverse events

As argued above, checkpoint blockade lowers a tolerance threshold behind which every patient sits at a different distance. The question here is what sets that distance. The determinants fall into four groups that differ in how fixed they are and how early they can be measured: germline genetics, fixed at birth; the immune landscape, partly measurable at baseline and partly emerging on treatment; the microbiome and metabolic state, modifiable and environmentally shaped; and clinical characteristics, present in every patient record today (Table 1). One theme recurs across all four and is worth stating in advance. Many of the same host features that raise the risk of toxicity also predict a better antitumour response. Susceptibility to irAEs is not simply a safety liability to be screened out. It is frequently entangled with the biology of benefit, which makes host stratification a problem of optimising a trade-off rather than minimising a harm.

**TABLE 1:** Major host determinants of immune-related adverse events, with stage and evidence level.

Category	Determinant	Reported association with irAEs	Stage	Also predicts response?	Evidence level	Refs
Germline genetics	IL7 locus (rs16906115)	∼2.1× higher all-grade irAE risk across 12 cancer types	Pre-treatment	Yes — improved melanoma survival	GWAS, replicated	Groha et al., 2022, Taylor et al., 2022
Germline genetics	HLA-DRB1 shared epitope/DRB1*04:05	ICI inflammatory arthritis (≥1 SE allele 61.5% vs. 41.2%; OR 2.3)	Pre-treatment	—	Case–control	Cappelli et al., 2019
Germline genetics	HLA class II (DRB1*04:05, DPB1*05:01)	ICI-induced type 1 diabetes	Pre-treatment	—	Small cohort	Inaba et al., 2022
Germline genetics	HLA-DR alleles (DR4, DR15, DR11)	Organ-specific irAEs: T1DM, hypophysitis, pneumonitis	Pre-treatment	—	Cohort; ethnicity-dependent	Akturk et al., 2022
Germline genetics	CTLA4/PDCD1/cytokine-gene SNPs; polygenic risk scores	Candidate associations; PRS an emerging route	Pre-treatment	—	Candidate/exploratory	Chin et al., 2022
Baseline immune	Activated CD4 memory T cells + TCR diversity	Severe irAE, independent of organ system	Pre-treatment	Entangled	Multi-cohort discovery	Lozano et al., 2022
Immune (dynamic)	≥30% drop in circulating B cells + ≥2× rise in CD21Lo B cells/plasmablasts	High-grade irAE, ∼3 weeks before onset	On-treatment	—	Prospective, small	Das et al., 2018
Immune	Pre-existing/treatment-emergent autoantibodies (e.g., anti-thyroid)	Organ-matched toxicity	Pre-treatment/on-treatment	—	Cohort-level	de Moel et al., 2019
Microbiome	Faecalibacterium/Firmicutes-rich gut flora	Higher colitis incidence	Pre-treatment	Yes — better response/survival	Prospective, small	Chaput et al., 2017
Microbiome	Bacteroidetes-dominant gut flora	Resistance to colitis	Pre-treatment	—	Prospective, small	Dubin et al., 2016
Microbiome	Antibiotic exposure at initiation	Modifier; disrupts protective communities	Peri-treatment	—	Observational	Schneider et al., 2026
Metabolic	Obesity/high BMI	Proposed modifiers; weak and inconsistent	Pre-treatment	—	Hypothesis-generating	Zhang et al., 2021
Clinical	Pre-existing autoimmune disease	∼75% flare, *de novo* irAE, or both; worse if active disease	Pre-treatment	—	Systematic review	Abdel-Wahab et al., 2018, Menzies et al., 2017

SE, shared epitope. “Entangled” = the feature is mechanistically tied to response but not reported as an independent response predictor. “Evidence level” reflects study design and maturity, not formal quality grading; no determinant is prospectively validated for clinical decision-making.

### 3.1 Genetic predisposition

Germline genetics is the only host determinant fixed before the first dose, and therefore the most attractive for pre-treatment prediction. The strongest single-variant evidence comes from a genome-wide association study of 1,751 patients treated with ICIs across 12 cancer types, which identified a common variant near IL7 (rs16906115) associated with all-grade irAEs, with a hazard ratio of roughly 2.1 and replication in independent cohorts (Groha et al., 2022). The signal is mechanistically coherent: the risk allele produces a B-cell-specific regulatory effect on IL7, a central regulator of lymphocyte homeostasis, and carriers show greater lymphocyte stability after ICI initiation (Groha et al., 2022; Taylor et al., 2022). Critically, in a melanoma cohort the same risk allele that increased severe irAE risk independently associated with improved survival (Taylor et al., 2022), the review’s recurring duality in its cleanest form: a single inherited variant that raises both toxicity and benefit.

The most developed body of genetic evidence, however, concerns the human leucocyte antigen (HLA) locus, and it carries a lesson that reaches beyond risk prediction into the question of organ specificity taken up later. Because HLA molecules determine which self-peptides are presented to T cells, HLA genotype is the most direct germline route by which a host can bias toxicity toward one organ rather than another, and the reported associations behave accordingly: they are largely organ-specific, and they tend to mirror the HLA associations of the corresponding spontaneous autoimmune disease. Checkpoint-induced inflammatory arthritis is enriched for the HLA-DRB1 shared-epitope alleles that define seropositive rheumatoid arthritis risk, with at least one shared-epitope allele in 61.5% of affected patients versus 41.2% of controls (odds ratio 2.3), and an excess of DRB1*04:05 specifically (Cappelli et al., 2019). Checkpoint-induced type 1 diabetes recapitulates classical diabetes genetics, with reported enrichment of DRB1*04:05, DPB1*05:01 and related class II alleles (Inaba et al., 2022). The clearest demonstration comes from a melanoma cohort genotyped at HLA-DR, in which distinct DR alleles were each associated with a distinct tissue-specific irAE, confirming earlier single-toxicity reports of DR4 with type 1 diabetes, DR15 with hypophysitis and DR11 with pneumonitis (Akturk et al., 2022). The same principle extends beyond melanoma, with HLA genotype associated with irAE risk in non-small-cell lung cancer (Abed et al., 2022). This is molecular evidence for the review’s central claim: a host-encoded, germline factor that helps select not merely whether toxicity occurs but where. The caveat is that these associations are cohort-specific, strongly ethnicity-dependent, and not uniformly replicated. A melanoma study of HLA-A*02:01, for instance, found no convincing association with organ-specific irAEs (Manger et al., 2025).

Beyond HLA, candidate associations have been reported for polymorphisms in the checkpoint genes themselves (CTLA4, PDCD1) and in cytokine-pathway genes, and polygenic risk scores that aggregate many small-effect variants are the emerging route to convert these scattered signals into a usable pre-treatment test (Chin et al., 2022). The appeal of germline prediction is that it is stable, measurable once, and available before any exposure. Its limitations are that individual variants explain only a fraction of risk, and that most associations still await prospective, ancestry-diverse validation.

### 3.2 Baseline immune landscape

The immune system a patient brings to treatment is the substrate on which checkpoint blockade acts, but a distinction has to be drawn at the outset that the literature often blurs: whether a marker is measured before the first dose or only after treatment has begun. That distinction determines what the marker can be used for. Genuine pre-treatment markers can triage patients before any exposure; on-treatment changes can only flag toxicity that is already developing. Both are useful, but they belong to different clinical stages, and several of the strongest immune-landscape signals fall into the second category rather than the first.

The best-characterised example is in the B-cell compartment. In patients receiving combination CTLA-4 and PD-1 blockade, a fall of at least 30% in total circulating B cells together with a two-fold or greater rise in CD21lo B cells and plasmablasts after a single cycle identified those who developed high-grade irAEs, a median of 3 weeks before clinical onset (Das et al., 2018). This is a powerful signal, but it is an early-on-treatment change rather than a baseline feature. It is a surveillance marker, not a triage one. Autoantibodies span both stages: pre-existing autoantibodies are a true baseline signal, and their specificity often predicts the target organ, with thyroid autoantibodies concentrated in patients who develop thyroid irAEs, whereas treatment-emergent autoantibodies are again an on-treatment readout (de Moel et al., 2019). A wider set of peripheral-blood correlates has been reported, including baseline and early cytokine profiles integrated into composite toxicity scores (Lim et al., 2019), the neutrophil-to-lymphocyte ratio and related routine blood indices (Wang et al., 2024), eosinophil counts, and T-cell repertoire diversity, distributed similarly across the baseline and on-treatment divide.

Two caveats follow. Most of these signals still await prospective validation in independent cohorts. And, more consequentially for a review about prediction, the immune landscape is where the line between predicting toxicity and merely detecting its earliest phase is thinnest, which is exactly why the baseline-versus-on-treatment distinction must be made explicitly rather than left implicit. It structures the prediction framework developed under precision prediction: baseline determinants for triage before treatment, dynamic immune markers for surveillance once it has started.

### 3.3 Microbiome and metabolic factors

Unlike genetics and, largely, the immune landscape, the microbiome and metabolic state are modifiable, which makes them the host determinants most interesting as intervention points. The gut microbiome has the firmest evidence, concentrated on ICI colitis. In metastatic melanoma treated with ipilimumab, a baseline microbiota enriched for Faecalibacterium and other Firmicutes was associated with a higher incidence of colitis, whereas a Bacteroidetes-dominant community was associated with resistance to it (Chaput et al., 2017; Dubin et al., 2016). The proposed mechanism runs through short-chain fatty acids and their support of regulatory T-cell tone at the mucosal barrier. The duality appears here too: the same Firmicutes-rich, colitis-prone configuration was associated with better response and survival (Chaput et al., 2017), so a microbiome that protects the gut may also blunt the tumour. Antibiotic exposure around the start of therapy, which disrupts exactly these communities, is a related and clinically common modifier (Schneider et al., 2026). The metabolic determinants are more tentative. Higher body-mass index has been associated with an increased incidence of irAEs in pan-cancer cohorts (Zhang et al., 2021). Type 2 diabetes, dyslipidaemia, ageing, and vitamin D status have each also been proposed to modify risk, but the evidence is thinner, less consistent, and confounded by their effects on response and on baseline inflammation, and it should be read as hypothesis-generating rather than established.

### 3.4 Patient-specific clinical characteristics

Clinical characteristics are the determinants already in every patient’s chart, requiring no assay, and they remain the practical baseline against which molecular predictors must prove added value. The best-supported is pre-existing autoimmune disease. A systematic review of patients with autoimmune disease treated with ICIs found that roughly three-quarters experienced a flare of their underlying condition, a *de novo* irAE, or both (Abdel-Wahab et al., 2018), and flares are more frequent in those with active rather than quiescent disease at the start of therapy (Menzies et al., 2017). This is a mechanistically expected result: a patient whose peripheral tolerance is already compromised has less margin to lose. Other clinical variables, including age, sex, body-mass index (Zhang et al., 2021), tumour type, prior anticancer therapy, and chronic viral infection, are frequently cited as modifiers (Khoja et al., 2017), but the evidence for most is inconsistent across cohorts, with sex effects in particular reported in both directions. Their value is less as standalone predictors than as covariates any host-based model must account for.

### 3.5 Interactions among host determinants

These determinants are usually presented as a list, but they do not act independently, and treating them separately understates their predictive power. Germline variation helps shape both the baseline immune landscape and the composition of the microbiome. The microbiome in turn conditions systemic immune tone, and pre-existing autoimmune disease is itself partly genetic and is mirrored in the autoantibody repertoire. A patient’s position relative to the tolerance threshold is the joint product of these layers, not the sum of independent risk factors, which is why single markers have so far shown only modest predictive performance and why the field is moving toward integrated, multi-parameter models. Two consequences carry into the rest of the review. First, prediction is likely to require measuring several determinant classes together and combining them, which is taken up later. Second, because so many of these determinants predict response as well as toxicity, the aim of stratification cannot be to identify and exclude the susceptible; it must be to place each patient on a benefit-versus-toxicity gradient on which the same host features often push in the same direction. This duality rests specifically on pre-treatment host features being associated with both outcomes, such as the IL7 risk allele and Firmicutes-rich gut flora. It is distinct from the separate and immortal-time-confounded observation that patients who develop irAEs survive longer, and does not depend on it.

## 4 Multi-omics and biomarker discovery for risk stratification

As established above, host determinants act jointly, and single markers therefore predict poorly. Multi-omics is the technical response to that conclusion, a set of tools for measuring several layers of host biology at once and at high resolution (Table 2). The survey here covers what those tools are and, more importantly, what they have found. It is deliberately confined to measurement and discovery. The translation of these readouts into a decision at the bedside, a triage test before treatment or a monitoring schedule during it, is taken up later. One caveat frames everything that follows: almost all of this evidence comes from small, retrospective, single-institution cohorts, and little has been prospectively validated, so the appropriate reading is of a field identifying candidates rather than delivering tests.

**TABLE 2:** Multi-omics technologies and their applications in irAE research.

Technology	Measures	Application/key finding in irAEs	Sample	Refs
Single-cell RNA-seq	Per-cell transcriptomes	Clonal cytotoxic CD8 T cells dominate ICI colitis; pathology distinct from ulcerative colitis; epithelial–immune crosstalk	Affected tissue	Luoma et al., 2020, Thomas et al., 2024
scRNA-seq + single-cell TCR-seq	Transcriptome + clonality	Clonal cytotoxic effector-memory CD8 T cells in ICI myocarditis	Affected tissue	Zhu et al., 2022
Spatial transcriptomics/multiplex imaging	Cell position in tissue	Localises effector populations and epithelial interactions; still nascent and organ-limited for irAEs	Affected tissue	Thomas et al., 2024
Mass cytometry (CyTOF)	High-dimensional protein/cell states	Baseline activated CD4 memory abundance associated with severe irAE	Peripheral blood	Lozano et al., 2022
Bulk + single-cell TCR-seq	Clonal repertoire structure	Baseline TCR diversity and on-treatment clonal expansion predict/time irAE	Blood + tissue	Zhu et al., 2022, Lozano et al., 2022
Antigen-specific T-cell/TCR mapping	Antigen specificity	α-myosin (heart) and shared tumour–tissue antigens (NSCLC-skin) drive organ-specific irAE	Blood + tissue + tumour	Axelrod et al., 2022, Berner et al., 2019
Circulating proteomic/cytokine panels	Soluble mediators	Complementary, easily sampled layer; signals often on-treatment	Peripheral blood	Lim et al., 2019 *,* Wang et al., 2024
Machine learning/systems immunology	Integrates multiple layers	irAE-prediction models; limited by overfitting and absent external validation	Multi-source	Lozano et al., 2022, Kang et al., 2026

### 4.1 Transcriptomics and single-cell technologies

Bulk transcriptomics established that irAE-affected tissue carries inflammatory and interferon signatures, but the resolution to identify which cells drive the process came with single-cell RNA sequencing. Applied to the affected organ, it has been unexpectedly consistent across very different toxicities. In checkpoint-induced colitis, single-cell profiling identified clonally expanded, cytotoxic CD8 T cells as the dominant activated population, with a pathology distinct from ulcerative colitis (Luoma et al., 2020), a picture refined by a later multi-omic analysis of roughly 300,000 cells that mapped crosstalk between circulating and tissue-resident immune cells and the colonic epithelium (Thomas et al., 2024). In checkpoint-induced myocarditis, one of the most lethal irAEs, single-cell and paired T-cell-receptor (TCR) sequencing found the same signature in a different organ: clonally expanded, cytotoxic effector-memory CD8 T cells re-expressing CD45RA (Zhu et al., 2022). The recurrence of a clonally expanded, cytotoxic CD8 programme across colon and heart is one of the more important observations in the field, because it suggests a shared effector grammar underlying organ-specific toxicities, a point taken up later. The limitation is equally clear: these are studies of tissue already inflamed, so they illuminate mechanism far better than they predict risk.

### 4.2 Spatial biology and proteomics

Dissociated single-cell methods lose the one piece of information that organ-specific toxicity most depends on, which is where the cells are. Spatial and imaging-based approaches restore it. The multi-omic colitis work above localised its populations in tissue and identified epithelial interactions that a suspension assay would miss (Thomas et al., 2024), and multiplexed imaging and spatial transcriptomics are beginning to be applied to irAE biopsies, though this literature is still nascent and organ-limited. On the protein side, high-dimensional cytometry has been the most productive tool: mass cytometry applied to peripheral blood resolves immune-cell states at a depth flow cytometry cannot, and was central to identifying the circulating T-cell populations discussed below (Lozano et al., 2022). Circulating proteomic and cytokine panels add a complementary, easily sampled layer, though their signals, as noted earlier, are frequently on-treatment rather than baseline.

### 4.3 Immune repertoire profiling

T-cell-receptor sequencing measures the clonal structure of the T-cell response, and it has produced signals at both stages of the two-stage frame. At baseline, higher pretreatment TCR diversity, together with a greater abundance of activated CD4 memory T cells, was associated with severe irAE development across organ systems in a multi-cohort melanoma study (Lozano et al., 2022). On treatment, the clonal expansion seen in affected tissue, and the correlation between early circulating clonal expansion and the timing of irAE onset, provide a dynamic readout (Zhu et al., 2022; Lozano et al., 2022). Repertoire data also surface the review’s duality most sharply, because clonal expansion of cytotoxic T cells is simultaneously a mechanism of antitumour response and of toxicity. The analytical challenge is to distinguish the clones driving tumour regression from those attacking normal tissue, which current repertoire methods cannot yet do reliably.

### 4.4 Artificial intelligence and systems immunology

The determinants and data types above are numerous, high-dimensional, and interacting, which is precisely the setting in which machine learning is useful. Models integrating immune signatures have been trained to predict irAEs, for example, one built on interferon-primed immune-landscape features across several algorithms (Kang et al., 2026), and systems-immunology approaches attempt to combine germline, cellular, and repertoire layers into a single predictor. The promise is real, but so are the failure modes, and they are the familiar ones: small sample sizes relative to feature counts, a strong risk of overfitting, and a near-absence of external validation. A model that reports high cross-validated accuracy in a single hundred-patient cohort and is never tested elsewhere is a hypothesis, not a test.

### 4.5 Integrated biomarker models for toxicity prediction

The logical endpoint here is a model that combines several determinant classes into one risk estimate. The clearest existing example integrated the baseline cellular and repertoire features above into a composite score that predicted severe irAE risk and showed some ability to distinguish irAE grade (Lozano et al., 2022). This is a proof of concept for integration, not a deployable instrument: the cohorts are small, the models retrospective, and prospective, multi-site validation is largely absent. That gap, and what it would take to close it, belongs to the discussion that follows, which turns from what can be measured to what could be done with it. The measurement layer is maturing quickly; the evidence that any integrated model changes a treatment decision safely does not yet exist.

## 5 Host determinants of organ-specific immune toxicity

The preceding discussion treated irAE risk largely as a single quantity: how likely a patient is to develop toxicity of any kind. But the clinical reality is organ-specific. One patient on anti-PD-1 develops thyroiditis, another on the same drug develops pneumonitis, a third develops nothing. Explaining that divergence is the hardest problem in the field, and it is where the temptation to overclaim is strongest. The discussion here resists two opposite errors. The first is to treat organ specificity as drug-determined alone; the second is to treat it as host-determined alone. It is neither. Organ specificity is the product of a drug-set baseline and host factors layered upon it (Figure 1), and the discussion is organised around that interaction.

**FIGURE 1:**
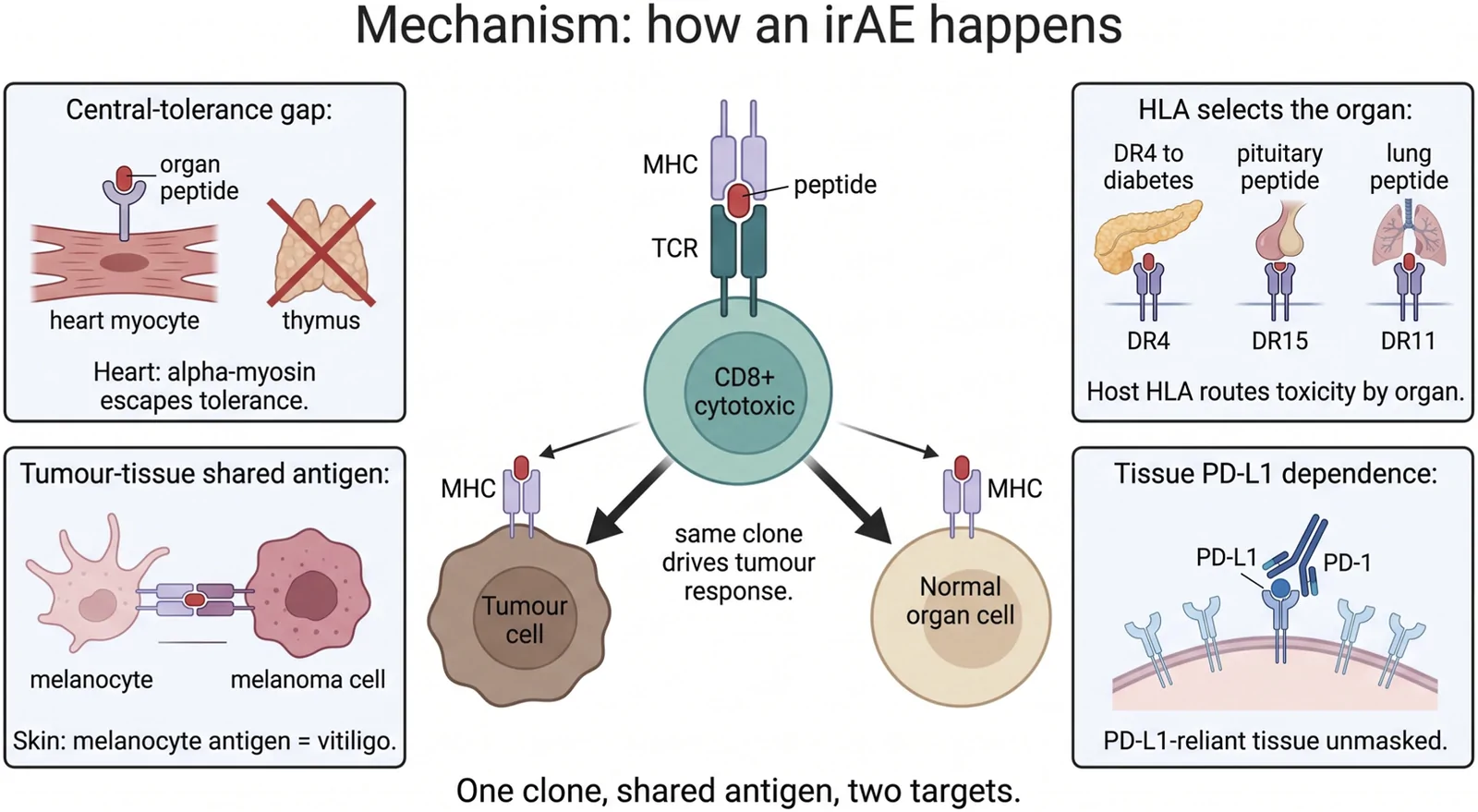
Organ-specific immune-related toxicity as a host-gated, shared-antigen mechanism. Centre: a cytotoxic CD8 T-cell clone recognises a peptide (magenta) shared by a tumour cell and a normal organ cell, so one clone attacks both, and the response that damages the organ also drives antitumour activity. Surrounding panels show the host factors that select which organ is hit: organ antigens that escaped thymic tolerance (e.g., alpha-myosin in heart), the HLA genotype that routes toxicity by presenting organ-specific peptides (DR4 pancreas, DR15 pituitary, DR11 lung), tumour-tissue shared antigens (melanocyte antigens linking melanoma response to vitiligo), and tissue reliance on local PD-L1. Checkpoint blockade releases the T-cell brake but does not select the organ; host determinants do. Figure was Created in BioRender. Alwithenani, A. (2026) https://BioRender.com/63n3npu.

### 5.1 Why different patients develop different irAEs

The starting point is not the host but the drug. As established earlier, the checkpoint blocked sets a baseline organ distribution: anti-CTLA-4 skews toward colitis and hypophysitis, anti-PD-1 toward pneumonitis, thyroid dysfunction and vitiligo (Khoja et al., 2017). This class signature is the menu of likely toxicities. What host factors determine is which item off that menu a given patient receives. The evidence that the selection is host-encoded has been assembled earlier and converges here. HLA genotype maps to specific target organs, with distinct DR alleles associated with distinct tissue-specific irAEs and each tending to mirror the HLA association of the corresponding spontaneous autoimmune disease (Cappelli et al., 2019; Inaba et al., 2022; Akturk et al., 2022). Pre-existing organ-directed autoantibodies predict the matching organ toxicity, as with thyroid autoantibodies and thyroiditis (de Moel et al., 2019). And the cleanest experimental demonstration remains the one introduced earlier: in PD-1-deficient mice, the identical genetic lesion produces lupus-like nephritis on one genetic background and autoimmune cardiomyopathy on another, the checkpoint setting the stage and host genotype selecting the organ (Nishimura et al., 1999; Nishimura et al., 2001). Drug class defines the possible; host defines the actual.

### 5.2 Tissue-specific immune microenvironments

Organs are not equally exposed to a given checkpoint blockade, because they differ in how much they depend on that checkpoint for their own tolerance. PD-1 and its ligand act mainly in peripheral tissues, where PD-L1 expressed on parenchymal and stromal cells restrains activated T cells locally (Keir et al., 2008). Tissues that rely most heavily on this local brake are, predictably, the ones most exposed when it is released, which is part of why the anti-PD-1 organ signature falls where it does. The affected-tissue single-cell studies described earlier add the cellular substrate: the dominant effector populations in irAE lesions are often tissue-resident, and the colonic and cardiac infiltrates are shaped by local chemokine and epithelial cues rather than assembled from scratch (Luoma et al., 2020; Thomas et al., 2024). The shared cytotoxic-CD8 programme identified across organs supplies the effector mechanism, but the tissue microenvironment, its resident clones, its antigen display and its chemokine milieu, determines where that program lands.

### 5.3 Organ susceptibility and immune tolerance

Why is a particular organ vulnerable at all? The most complete answer ties organ specificity directly back to the tolerance architecture described earlier. An organ is susceptible when it harbours a self-antigen that escaped central tolerance and can be presented by the host’s HLA, so that anti-self clones were never deleted and sit waiting for the peripheral brake to lift. Checkpoint-induced myocarditis is the paradigm. Its dominant clonal CD8 T cells recognise α-myosin, a cardiac protein that is absent from the thymus and therefore never subject to thymic negative selection. The anti-α-myosin clones persist in every individual and are unleashed when checkpoint blockade removes the peripheral restraint that had held them (Axelrod et al., 2022). Organ susceptibility, on this account, is highest where central tolerance is weakest, which is exactly where the earlier argument predicted the threshold would sit closest.

### 5.4 Mechanistic insights into heterogeneous clinical manifestations

The threads converge on a single mechanism: shared antigens between the tumour and normal tissue. Because checkpoint blockade amplifies T cells against tumour antigens, any normal organ expressing an antigen the tumour also carries becomes collateral. In non-small-cell lung cancer, the organ struck by toxicity is the one most antigenically similar to the tumour, with skin ranking second only to lung, and identical T-cell-receptor sequences are found in the primary tumour and the inflamed skin (Berner et al., 2019). In melanoma, the shared melanocyte differentiation antigens MART-1, gp100 and the tyrosinase-related proteins are targeted in both the tumour and normal melanocytes, so the same response that regresses the tumour depigments the skin as vitiligo (Hua et al., 2016). This is the review’s duality at its most mechanistic and most literal: not merely that toxicity and benefit correlate, but that a single antigen-specific clone executes both, which is why vitiligo in melanoma and skin toxicity in lung cancer track with response rather than opposing it (Berner et al., 2019; Hua et al., 2016).

Assembled, these pieces yield a model of organ specificity with no single author. The target organ is selected by the intersection of the drug class, which sets the compartment and stage of the released response (Khoja et al., 2017); the host’s HLA, which determines which self-peptides can be presented (Cappelli et al., 2019; Inaba et al., 2022; Akturk et al., 2022); the gaps in central tolerance for particular organ antigens, which determine which anti-self clones survived (Axelrod et al., 2022); the antigens the tumour happens to share with normal tissues, which determine where a productive antitumour response spills over (Berner et al., 2019; Hua et al., 2016); and the tissue’s own dependence on the blocked checkpoint for local tolerance (Keir et al., 2008). Organ specificity is neither a property of the drug nor of the patient but of their intersection, and this is why predicting not merely whether but where toxicity will strike remains unsolved. That unsolved problem is the one taken up next.

## 6 Toward precision prediction and personalised immunotherapy

Up to this point, the review has described what host biology can be measured and why toxicity localises where it does. What follows asks what could be done with that knowledge in the clinic. The honest starting point is how little is done now. Current standard of care is almost entirely reactive: the major clinical guidelines address how to recognise and treat irAEs once they have occurred, not how to predict them beforehand, and no host-based pre-treatment predictor is in routine use (Brahmer et al., 2021). The aim of a precision approach is to move upstream of the event, and the two-stage structure introduced earlier, baseline triage before treatment and dynamic surveillance during it, organises how that might work.

### 6.1 Risk prediction before treatment

The first stage uses determinants measurable before the first dose. The review has assembled the candidates: germline variants such as the IL7 locus and the organ-directing HLA alleles (Groha et al., 2022; Taylor et al., 2022; Akturk et al., 2022), the baseline circulating immune state, in particular activated CD4 memory T-cell abundance and TCR diversity (Lozano et al., 2022), gut microbiome composition (Chaput et al., 2017; Dubin et al., 2016), and clinical history led by pre-existing autoimmune disease. None of these is yet a validated clinical test, and individually each is only modestly predictive, which is the expected consequence of the interactions described earlier. The realistic path is the integrated, multi-parameter model discussed earlier, evaluated prospectively rather than in the retrospective single cohorts that dominate today. In the nearer term, the more achievable use of baseline risk is not a binary treat-or-not decision but the tuning of choices already in the clinician’s hands: a patient predicted to be at high risk might be directed toward monotherapy rather than combination blockade, which roughly triples the rate of grade 3 or 4 events (Larkin et al., 2015), and might enter treatment under a more intensive monitoring schedule.

### 6.2 Precision monitoring during therapy

The second stage uses the on-treatment markers separated earlier from true baseline signals. An early fall in circulating B cells with a rise in CD21lo cells and plasmablasts flags high-grade risk weeks before clinical onset (Das et al., 2018); early circulating T-cell clonal expansion tracks the timing of that onset (Lozano et al., 2022); and treatment-emergent autoantibodies signal the organ under attack (de Moel et al., 2019). These markers do not prevent an irAE, but they can compress the interval between the immunological event and its clinical recognition, which is where much of the morbidity of severe irAEs is generated. The two stages are complementary rather than competing: baseline determinants set pre-treatment risk and the intensity of surveillance, and on-treatment markers catch the emerging event within that surveillance.

### 6.3 Individualized prevention

Prevention, as distinct from the management of established toxicity that this review has deliberately left to existing guidelines (Brahmer et al., 2021), follows from prediction in three ways. The first is regimen selection by predicted risk, as above. The second concerns the modifiable determinants: avoiding unnecessary antibiotics around the initiation of therapy protects the microbial communities associated with lower colitis risk, and the prospect of deliberate microbiome modulation is raised by proof that the axis is causal. Faecal microbiota transplantation can resolve checkpoint colitis refractory to immunosuppression (Wang et al., 2018), which establishes that the gut microbiome is not merely correlated with toxicity but causally involved and modifiable. Its current application is the treatment of an established event, and its extension to prophylaxis in predicted-high-risk patients is untested. It also carries the review’s recurring hazard, since a microbial state that protects the gut may also protect the tumour. The third is targeted baseline screening: a patient whose HLA type marks elevated risk for a specific organ toxicity can be given organ-directed baseline assessment and closer surveillance of that system, turning the organ-specific associations described earlier into a concrete monitoring plan.

### 6.4 Future directions in precision immuno-oncology

Two problems define the frontier. The first is that almost every predictor discussed forecasts whether a patient will develop toxicity, not where. As shown earlier, organ selection is legible in principle, through HLA restriction and tumour-tissue antigen sharing, but no predictor yet turns that legibility into organ-specific forecasting. Doing so is the harder and more clinically valuable target. The second is the duality that has recurred throughout: because host determinants predict response and toxicity together, a predictor must be judged on both axes at once. A model that reduces toxicity by selecting out the patients who would also have responded has failed, even if its toxicity numbers improve. This reframes the objective from minimising harm to optimising the benefit-to-toxicity ratio, and it means predictive models should be validated against response as well as against irAEs. A recently proposed conceptual framework reinforces the benefit-to-toxicity argument presented in this review. By drawing an analogy with allogeneic transplantation, it has been suggested that, under checkpoint blockade, a patient’s own T cells may exhibit behaviour similar to the alloreactive responses observed in chronic graft-versus-host disease (cGVHD), resulting in a pattern of tissue injury that closely resembles this well-characterised transplant complication (Bakacs and Anderson, 2026). This parallel extends beyond descriptive comparison; comparative transcriptomic analyses demonstrate that cGVHD and ICI-induced irAEs converge on a shared, interferon-conditioned tissue-state ecosystem. This observation aligns with the interferon signatures and clonal cytotoxic CD8 programme identified across irAE-affected organs in the single-cell studies discussed in Section 4.1. This conceptualisation has direct translational implications that support the central claim of this review: if much of the toxicity arises from an exaggerated alloreactive-like response, it may be possible to attenuate this response without abolishing antitumour immunity. Early clinical experience with ultra-low-dose ICI regimens supports this hypothesis, with reports indicating that meaningful antitumour activity can be maintained while toxicity is substantially reduced (Bakacs and Anderson, 2026). Dose-directed strategies complement the host-stratification approach advocated in this review, offering a means to optimise the benefit-to-toxicity balance through both patient selection and adjustment of drug regimen intensity. These strategies warrant prospective evaluation.

## 7 Conclusion and future perspectives

As a narrative synthesis, this review carries limitations that bear on how its conclusions should be read. Because sources were selected for conceptual relevance rather than through a systematic protocol, it is subject to selection and language bias, and studies reporting null associations may be under-represented. The evidence base is also a constraint: much of it comes from small, retrospective, single-institution cohorts with limited ancestral diversity, and few associations have been prospectively validated. These constraints reinforce rather than weaken the review’s central call for prospective, multi-site, ancestry-diverse validation. Collectively, the reviewed evidence demonstrates that the risk of irAEs is unevenly distributed among patients in ways that cannot be explained solely by the treatment regimen. The selection of a specific drug determines the maximum potential toxicity and influences which organs are most likely to be affected, while patient-specific factors ultimately determine who experiences the highest levels of toxicity. Within that envelope, the germline, the baseline immune landscape, the microbiome, and clinical history determine which patient crosses the tolerance threshold, and where. Three ideas carry the argument. First, pre-dose determinants and on-treatment dynamics address distinct clinical questions. Pre-dose determinants support triage, while on-treatment dynamics can only detect events that are already occurring. Conflating these concepts has led to an overestimation of the literature’s readiness for pre-treatment prediction. Second, the selection of one organ over another as a target is best explained at the drug-host interface, where HLA restriction and antigens shared between tumor and normal tissue represent the most tractable mechanisms. Third, and running through everything, the same host features that raise toxicity risk frequently raise the chance of response, so the clinical objective is not to screen out susceptible patients but to optimise a benefit-to-toxicity balance.

The candour of this review is that almost none of this is yet usable. No host-based pre-treatment predictor has been prospectively validated for clinical decision-making. The strongest single-marker associations explain only a fraction of risk, and the integrated models that should do better remain small, retrospective, and rarely tested outside their discovery cohort. Most predictors forecast whether toxicity will occur, not where, leaving the organ-specific question that matters most to patients largely unanswered. Much of the immune-landscape evidence is on-treatment rather than baseline, and much of the genetic evidence is drawn from populations of limited ancestral diversity, which constrains both generalisability and equity. Finally, the entanglement of toxicity and response means that any predictor validated against irAEs alone is only half-evaluated.

Progress depends less on new candidate markers, of which there are already many, than on the infrastructure to test them. The field needs prospective, multi-site, ancestry-diverse cohorts with mandated baseline and serial biospecimen collection, powered for the organ-specific endpoints that retrospective series cannot address. Integrated models should be built and then externally validated against both toxicity and antitumour response, so that a reduction in irAEs bought at the cost of efficacy is recognised as a failure rather than a success. Organ-specific prediction, anchored in HLA genotype and antigen-sharing, deserves particular effort because it is both the hardest problem and the one with the most direct clinical payoff. The assays that emerge will need a defined regulatory and companion-diagnostic path if they are to reach practice. The underlying biology reviewed here is sufficient to state the goal with confidence: a substantial part of who develops irAEs, and in which organ, is written in the host and is legible before treatment begins. Turning that legibility into a validated clinical tool, and with it moving checkpoint immunotherapy from the management of toxicity to its anticipation, is the work that remains.
